# Comparative Proteomics Combined with Morphophysiological Analysis Revealed Chilling Response Patterns in Two Contrasting Maize Genotypes

**DOI:** 10.3390/cells11081321

**Published:** 2022-04-13

**Authors:** Jinpeng Zou, Liang Yang, Yuhong Li, Mingxin Piao, Yaxing Li, Nan Yao, Xiaohong Zhang, Qian Zhang, Guanghui Hu, Deguang Yang, Zecheng Zuo

**Affiliations:** 1 College of Agriculture, Northeast Agricultural University, Harbin 150030, China; 17706314607@163.com (J.Z.); zhq2011@cau.edu.cn (Q.Z.); 2 Jilin Province Engineering Laboratory of Plant Genetic Improvement, College of Plant Science, Jilin University, Changchun 130062, China; 13311577331@163.com (L.Y.); piaomingxin@163.com (M.P.); 3 Basic Forestry and Proteomics Research Center, Fujian Agriculture and Forestry University, Fuzhou 350002, China; lyh317@163.com (Y.L.); fafu_lyx@163.com (Y.L.); 18844199632@163.com (N.Y.); hgsfzxh@163.com (X.Z.); 4 Institute of Maize Research, Heilongjiang Academy of Agricultural Sciences, Harbin 150030, China; gh_hu75@126.com

**Keywords:** maize, chilling stress, proteomics, differentially expressed protein, seedlings

## Abstract

Maize yield is significantly influenced by low temperature, particularly chilling stress at the maize seedling stage. Various physiological approaches have been established to resist chilling stress; however, the detailed proteins change patterns underlying the maize chilling stress response at the seedling stage remain unknown, preventing the development of breeding-based methods to resist chilling stress in maize. Thus, we performed comprehensive physiological, comparative proteomics and specific phytohormone abscisic acid (ABA) assay on different maize inbred lines (tolerant-line KR701 and sensitive-line hei8834) at different seedling stages (the first leaf stage and third leaf stage) under chilling stress. The results revealed several signalling proteins and pathways in response to chilling stress at the maize seedling stage. Meanwhile, we found ABA pathway was important for chilling resistance of tolerant-line KR701 at the first leaf stage. Related chilling-responsive proteins were further catalogued and analysed, providing a resource for further investigation and maize breeding.

## 1. Introduction

Environmental stress is a major factor causing significant crop yield losses, and a reduction in the productivity of crops of more than 70% annually has been reported to result from environmental stress [1,2,3]. Maize originating in tropical areas is highly sensitive to temperature changes, particularly to lower temperatures [4]. However, maize is widely accepted as a major economic and food crop, even in high-altitude areas [5]. Thus, maize often encounters chilling damage at the seedling stage and seed germination and seedling growth are seriously affected [6].

Current studies of chilling stress on plant are mainly focused on physiological responses, including agronomic traits related to maize morphology, physiology, and biochemistry during chilling stress [7,8]. These results suggest that chilling stress inhibits plant height and root length, causes obvious dwarfism, slows the growth of leaves and stems, decreases leaf numbers, inhibits leaf elongation [9,10,11,12], decreases root growth speed, and causes changes in metabolism and morphology [13,14,15]. Furthermore, chilling temperature increases lipid peroxidation in plant cells and then impairs the integrity of the cell membrane [16,17]. Additionally, chilling stress is correlated with reactive oxygen species (ROS), which affect the structure of cell membrane [18]. As reported previously, various ROS scavengers, including ascorbate peroxidase (APX), superoxide dismutase (SOD), catalase (CAT), peroxidase (POD), and glutathione reductase (GR), are synthesized by plants to improve the antioxidant defence ability and chilling resistance of plants [19]. With stronger chilling stress, maize plants show higher chlorophyll (Chl) activity, decreased Chl contents and more ROS induced by the increased relative leakage in the thylakoid membrane of leaves [20,21,22].

Previous studies further suggested that plants have various strategies to resist chilling stress, such as producing proline, flavonoids and trehalose, varying their growth patterns and regulating stomatal opening and closing [23,24,25], based on the related gene expression [16,26]. In previous studies, several genes and signal pathways responding to the chilling stress have been identified, such as cell wall synthesis, photosynthesis and metabolism pathways [23,27,28,29,30,31,32]. Additionally, several genes functioning in photosynthesis and the cell wall organization pathway also respond to chilling stress [33,34]. However, the mechanism of protein change patten by which plants resist chilling stress remains obscure, particularly in maize seedlings. Therefore, identifying the proteins and pathways responsive to chilling stress is critical to understanding the molecular mechanism of chilling resistance in maize seedlings. Previous omics studies of chilling stress mainly concentrated on transcriptomes [35,36,37,38,39,40]; however, the level of mRNA transcription is not necessarily consistent with that of the proteins associated with it, particularly during signal transduction [41,42]. Additionally, because protein is the functional unit of life activities [43], proteomics analysis is critical to identifying chilling responsive proteins (CRPs).

Several proteomics analyses have been previously performed on the chilling responses of maize [44,45,46]. Eighteen percent of proteins were changed between 20 and 16 °C in eighteen genetically diverse dent maize inbred-lines, antioxidative enzymes played an important role in maize anthers and detoxifying enzymes and antioxidants were used to scavenge ROS in maize line W9816 at the leaf stage under chilling stress. However, the mechanism by which maize seedlings resist chilling stress on a proteome scale remains unclear, and the temporal protein patterns and optimum period of maize development to defend against chilling stress are still not well defined. The seedling stage is a period severely affected by chilling stress; in this study, we performed quantitative proteomics analysis [47] of two maize inbred lines (chilling-tolerant-line KR701 and chilling-sensitive-line hei8834) at different seedling stages (first leaf and third leaf stages) under chilling stress. We analysed the proteomics data using two different algorithms (CRPs for individual proteins and WGCNA for modules). Interestingly, both proteome patterns formed with the two distinct algorithms suggested that hormone-mediated signalling pathways, particularly the ABA signalling response, are important for maize to resist chilling stress at the first leaf stage. The study also identified candidate CRPs and pathways functionally related to the chilling response of maize, providing a good resource for maize breeding in future.

## 2. Materials and Methods

### 2.1. Plant Materials and Stress Treatments

The maize inbred lines KR701 and hei8834, selected from 30 maize inbred lines, were used in this study [39]. To eliminate the effect of photoperiod on the expression of related proteins and ensure consistent growth conditions, maize seedlings were grown in a constant temperature incubator (HiPoint,740 FLED) at 25 °C (all day) with a 24 h photoperiod (continuous light) until the first leaf stage (7-d-old plants) and third leaf stage (12-d-old plants) [48,49,50].

The different chilling stress treatments were performed at the first leaf stage and third leaf stage, respectively: −chilling (control): 7-d-old plants and 12-d-old plants were grown at normal condition for 24 h. +chilling (chilling stress): 7-d-old plants and 12-d-old plants were grown at 4 °C for 24 h [9].

### 2.2. Physiological Analysis of Chilling-Treated Maize Seedlings

Agronomic traits: whole seedlings with different stress treatments were prepared and the plant height and root length were measured [12,51,52]. Physiological and biochemical traits: whole seedlings with different stress treatments were prepared and all traits (relative leakage, relative water content (RWC), Chl content, Fv/Fm, MDA content, SOD activity, POD activity) were measured as described previously [53].

### 2.3. Proteomics Analysis

An amount of 0.5 g of mixed maize samples (whole plant) was extracted with protein lysis buffer as described previously [54]. One hundred micrograms of protein from each seedling sample was digested for each repeat using the filter-aided sample preparation (FASP) method [55]. Then, the peptides were labelled using TMT10-plex kits (Thermo Fisher Scientific, Torrance, CA, USA) [56]. Digested peptides were fractionated using an Ultimate 3000 system (Thermo Fisher Scientific, Waltham, MA, USA) [57]. Peptide fractions were analysed by online nanospray LC–MS/MS on an Orbitrap Fusion coupled to an EASY-nano-LC system (Thermo Scientific, Waltham, MA, USA) [58]. Proteome Discoverer software 2.0 (Thermo Fisher, Shanghai, China) [59] was used to process the raw MS/MS data obtained. All the MS/MS samples were analysed using Sequest (Thermo Fisher Scientific, San Jose, CA, USA; Version 2.1.1.21), which was set up to search Zea_mays.AGPv3.22.pep.all.fasta (https://www.maizegdb.org/, 57,882 entries) (accessed on 7 July 2020). Scaffold Q^+^ (version Scaffold_4.7.1; Proteome Software Inc., Portland, OR, USA) [60] was used for protein identification.

### 2.4. Hormonal Analysis

The quantification of endogenous ABA was performed using an LC–MS/MS platform as described previously [61]. Plant materials (0.2 g FW) were frozen in liquid nitrogen, ground into powder, and extracted with 1 mL of methanol/water/methyl tert-butyl ether (1:3:1, *v*/*v*/*v*) at 4 °C. The extract was vortexed for 10 min and incubated in a cold ultrasonic bath for 10 min. Next, the extract was extracted with 650 μL of methanol/water (1:3, *v*/*v*) at 4 °C for 5 min and centrifuged at 14,000 rpm at 4 °C for 5 min. The aqueous extracts were evaporated to dryness and reconstituted in 50% methanol (*v*/*v*) before LC–MS/MS analysis. The raw data were imported to MS-DIAL 4.12 [62]. The data matrix was exported after peak extraction, denoising deconvolution and peak alignment. The positive hit results were compared using the databases MassBank, Respect, and GNPS in the MS and MS/MS information modes. Ten fully expanded leaves from 10 independent plants subjected to each of the stress treatments and control were pooled as one biological replicate for hormone quantification, and three biological replicates were performed.

### 2.5. GO Enrichment Analysis

GO Enrichment Analysi were performed using Blast2GO software (http://www.balst2go.org/version 5.1.13) (accessed on 20 July 2020) [63]. For enrichment analysis, Fisher’s exact test [64] was performed.

### 2.6. WGCNA

The WGCNA R package [65] was used to build coexpression networks. These networks comprised 6631 proteins. The R software package can be found at http://www.genetics.ucla.edu/labs/horvath/CoexpressionNetwork/Rpackages/WGCNA (accessed on 7 September 2020) [66].

### 2.7. Quantification and Statistical Analysis

All statistical data were collected in GraphPad Prism 8.0.2 (GraphPad Software, San Diego, CA, USA). ANOVA with two-tailed Student’s *t* test [67]. ^ns^ *p* > 0.05, * *p* < 0.05, ** *p* < 0.01, and *** *p* < 0.001. All the data were reported as means ± SD.

## 3. Results

### 3.1. Chilling Resistance Analysis of Inbred Lines at Different Maize Seedling Stages

**Figure 1:**
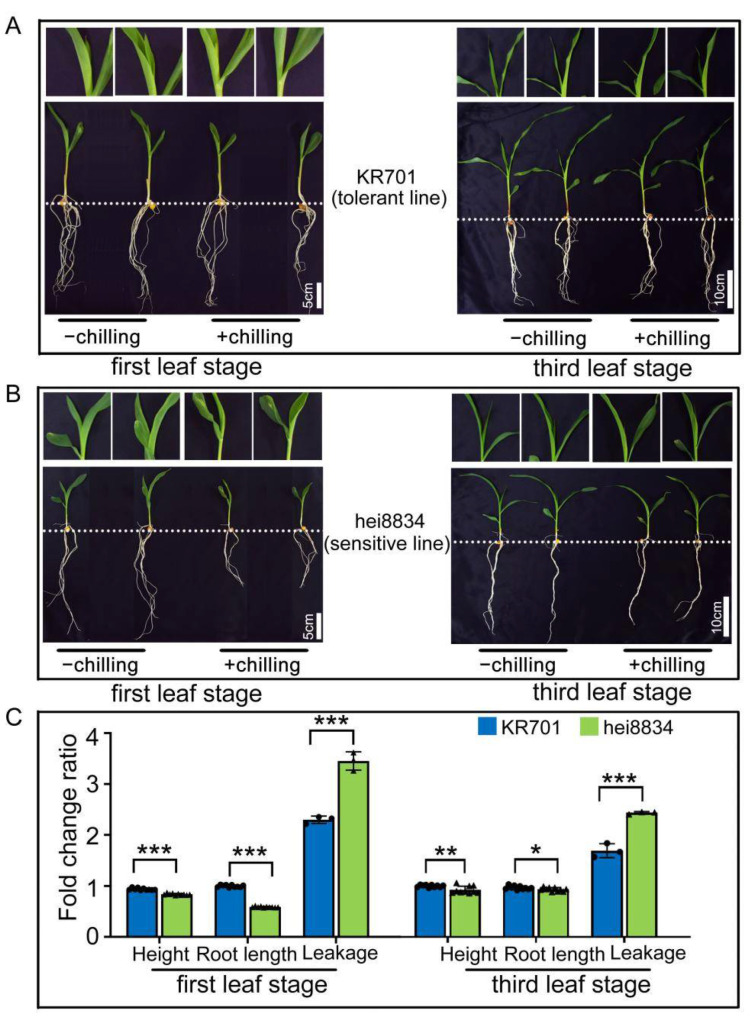
Physiological responses induced by chilling stress at different seedling stages. (**A**,**B**) Phenotypic changes in the inbred lines KR701 and hei8834 from the first leaf stage to the third leaf stage under normal condition and chilling stress condition. −chilling: uniformly growing seedlings were grown at normal condition for 24 h. +chilling: uniformly growing seedlings were grown at 4 °C for 24 h. (**C**) Fold change ratio of representative physiological traits (height, root length and relative leakage). Fold change ratio: +chilling/−chilling. Scale bar: 5 cm at the first leaf stage and 10 cm at the third leaf stage. The plant height and root length data are expressed as the means ± SD of 10 replicates. The “triangle”and “round” represented different repetitions. The relative leakage data are expressed as the means ± SD of three replicates. *, ** and *** denote levels of significance at *p* < 0.05, *p* < 0.01 and *p* < 0.001, respectively.

To analyse the different chilling resistances of maize inbred lines (KR701 and hei8834) with proteomics, we first physiologically examined these inbred lines in a laboratory environment. After chilling stress, the phenotypes and physiological and biochemical indicators were analysed in KR701 and hei8834 at the first leaf stage and third leaf stage, respectively. Compared to control (−chilling: maize seedlings grown at normal condition for 24 h), both KR701 and hei8834 inhibited leaf elongation and decreased plant height and root length in addition to leaf wilting under chilling stress (+chilling: maize seedlings grown at 4 °C for 24 h) (Figure 1A,B). However, compared with line hei8834, line KR701 showed faster leaf elongation, less serious leaf wilting and a lower degree of plant height and root length growth inhibition, indicating that KR701 has a greater chilling tolerance ability than hei8834. Furthermore, both inbred lines of maize seedlings at the first leaf stage were more vulnerable to chilling stress than those at the third leaf stage, suggesting that the first leaf stage is important for maize resistance to chilling stress. We next measured the height and root length of different inbred lines under the chilling stress. As shown in Figure 1C, Figures S1A,B and S2A,B and Table S1, compared to control, both the height and root length of KR701 and hei8834 were limited under chilling stress, whereas hei8834 showed more significant inhibition than KR701, particularly at the first leaf stage. Similar results were obtained from the relative leakage analysis, which is the most critical index to measure the response to chilling stress [68,69,70,71]. Compared to control, the relative leakage of KR701 and hei8834 both increased after chilling stress (Figure 1C, Figures S1C and S2C, Table S1), but a significant discrepancy was exhibited between KR701 and hei8834, in which hei8834 showed a significant increase in relative leakage compared with KR701. As expected, hei8834 still exhibited a stronger increase (1.5-fold) in relative leakage at the first leaf stage than KR701 (Figure 1C). Taken together, these physiological results suggested that the inbred line KR701 could be considered a chilling-resistant line compared with the inbred line hei8834. 

To analyse the different chilling resistances of maize inbred lines (KR701 and hei8834) with proteomics, we first physiologically examined these inbred lines in a laboratory environment. After chilling stress, the phenotypes and physiological and biochemical indicators were analysed in KR701 and hei8834 at the first leaf stage and third leaf stage, respectively. Compared to control (−chilling: maize seedlings grown at normal condition for 24 h), both KR701 and hei8834 inhibited leaf elongation and decreased plant height and root length in addition to leaf wilting under chilling stress (+chilling: maize seedlings grown at 4 °C for 24 h) (Figure 1A,B). However, compared with line hei8834, line KR701 showed faster leaf elongation, less serious leaf wilting and a lower degree of plant height and root length growth inhibition, indicating that KR701 has a greater chilling tolerance ability than hei8834. Furthermore, both inbred lines of maize seedlings at the first leaf stage were more vulnerable to chilling stress than those at the third leaf stage, suggesting that the first leaf stage is important for maize resistance to chilling stress. We next measured the height and root length of different inbred lines under the chilling stress. As shown in Figure 1C, Figures S1A,B and S2A,B and Table S1, compared to control, both the height and root length of KR701 and hei8834 were limited under chilling stress, whereas hei8834 showed more significant inhibition than KR701, particularly at the first leaf stage. Similar results were obtained from the relative leakage analysis, which is the most critical index to measure the response to chilling stress [68,69,70,71]. Compared to control, the relative leakage of KR701 and hei8834 both increased after chilling stress (Figure 1C, Figures S1C and S2C, Table S1), but a significant discrepancy was exhibited between KR701 and hei8834, in which hei8834 showed a significant increase in relative leakage compared with KR701. As expected, hei8834 still exhibited a stronger increase (1.5-fold) in relative leakage at the first leaf stage than KR701 (Figure 1C). Taken together, these physiological results suggested that the inbred line KR701 could be considered a chilling-resistant line compared with the inbred line hei8834. 

To further verify the above conclusion, six other chilling-related physiological and biochemical traits were measured after chilling stress, such as the MDA content, Chl content, RWC, SOD activity, POD activity and F_v_/F_m_ (Figures S1D–I and S2D–I, Table S1). The MDA content, SOD activity and POD activity under chilling treatment dramatically increased compared with those in the control group, and the values of Chl, RWC and F_v_/F_m_ decreased significantly. The fold change ratio indicated that all the traits described above were significantly decreased in hei8834 compared with those in KR701, except for the MDA content, which increased (Figures S1J and S2J, Table S1). These physiological and biochemical analyses also showed variation in maize at different periods of development under chilling stress.

### 3.2. Quantitative Proteomics Analysis of Maize Seedlings at Different Stages under Chilling Stress

**Figure 2:**
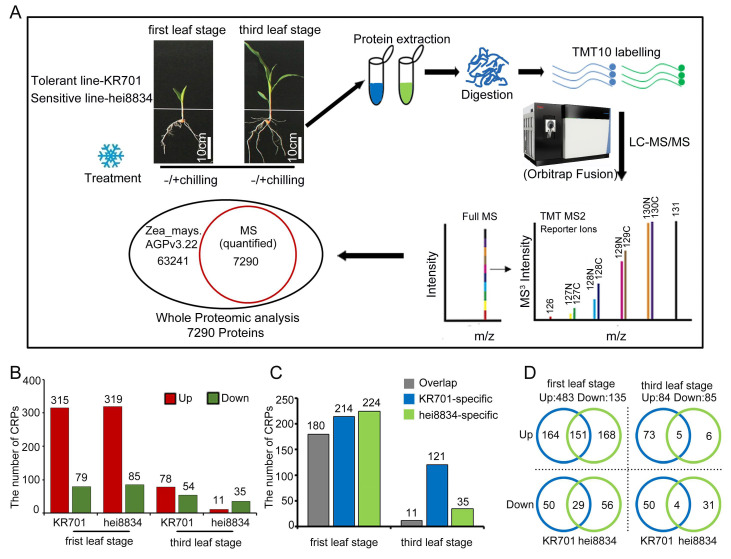
Quantitative proteomics analysis of chilling stress in maize seedlings. (**A**) Simple workflow for proteomics analysis. Experimental material backgrounds: tolerant- line KR701 and sensitive-line hei8834. Different stress treatments: −chilling: maize seedlings were grown at normal condition for 24 h. +chilling: maize seedlings were grown at 4 °C for 24 h. Diverse seedling stage: first leaf stage and third leaf stage. Scale bar: 10 cm. (**B**) Overall expression analysis of CRPs. (**C**) Stage-specific expression analysis of CRPs. (**D**) Specific classification analysis of CRPs.

Since the chilling-resistant line KR701 exhibited a different response than the chilling-sensitive-line hei8834 in resisting chilling stress, we performed combined proteomics quantification analysis to further investigate the discrepancies at the proteome level (Figure 2A, Table S2). A total of 62,753 peptides and 43,256 unique peptides were matched with the maize library; 8523 proteins were identified, and 7290 proteins were quantified (Figure S3A). The protein sequence coverage of 0–10%, 10–20%, 20–30%, 30–40%, 40–50%, 50–60% and >60% showed a consensus of 19.9%, 16.1%, 21.8%, 15.6%, 11.2%, 7.3%, 4.9% and 3.2%, respectively (Figure S3B). The size of most identified proteins was in the range of 20–80 kDa (Figure S3C). The distribution of peptides indicated that with increasing peptide number, the number of corresponding proteins decreased (Figure S3D). The *Pearson* correlation coefficient among the three biological replicates of proteomics was above 0.99, indicating consistency among our experimental results (Figure S4, Table S3). Principal component analysis (PCA) showed that the contribution ratios of principal components PC1 and PC2 were 40.1% and 29.4%, respectively, and the samples from various materials at different seedling stages were dispersed on PC1 and PC2, proving the diversity of the material and variability in the processing in our experiments (Figure S5A,B, Table S4).

The protein expression of 7290 proteins was quantified in all 8 samples, including in the different backgrounds (KR701 and hei8834), under different treatments (−chilling and +chilling) and at diverse seeding stages (the first leaf stage and third leaf stage) (Table S5). After comparing chilling proteomes, the fold change ratio of +chilling and −chilling >1.3 or <0.77 and *p* value of <0.05 were regarded as the CRPs. Based on these two criteria, 394 CRPs were quantified in KR701 and 404 in hei8834 at the first leaf stage. At the third leaf stage, the CRPs were significantly decreased in both KR701 and hei8834, 132 and 46 (only 34% and 11% of the first leaf stage), respectively (Figure 2B, Table S6). A scatter plot (Figure S5C–F) were used to classify and describe these CRPs, which better presented the distribution of the CRPs. Coexpression analysis showed that the distribution of CRPs varied between KR701 and hei8834. More importantly, a greater discrepancy was found between the first leaf and third leaf stages after chilling stress (Figure S6A).

### 3.3. Functional Analysis of CRPs

**Figure 3:**
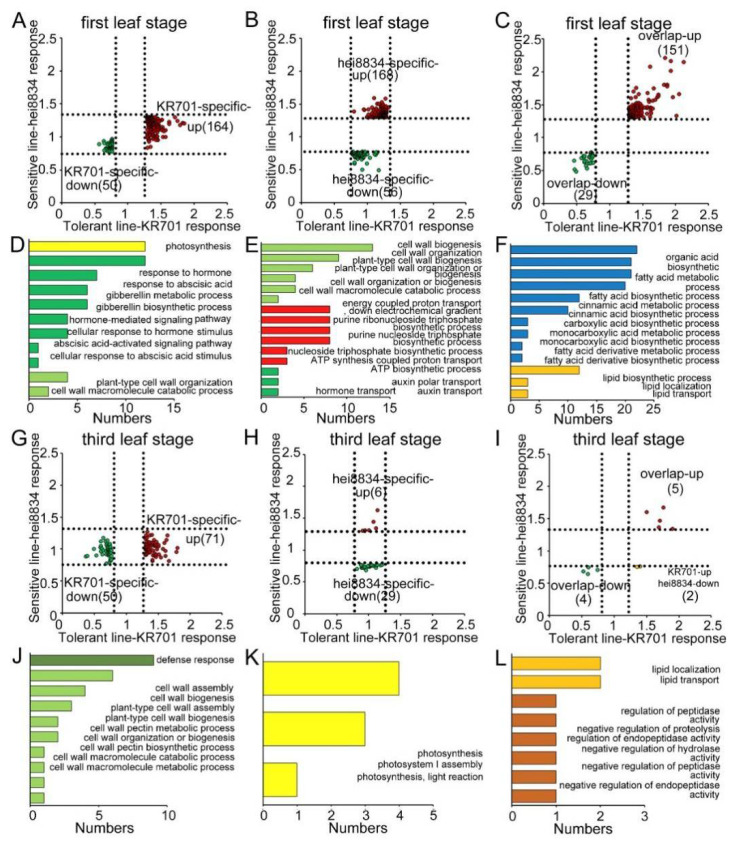
Distribution and GO functional enrichment analysis of CRPs. (**A**,**D**) Tolerant-line KR701-specific CRPs at the first leaf stage. (**B**,**E**) Sensitive-line hei8834-specific CRPs at the first leaf stage. (**C**,**F**) Overlapping CRPs of the tolerant-line KR701 and sensitive-line hei8834 at the first leaf stage. (**G**,**J**) Tolerant-line KR701-specific CRPs at the third leaf stage. (**H**,**K**) Sensitive-line hei8834-specific CRPs at the third leaf stage. (**I**,**L**) Overlapping CRPs of the tolerant-line KR701 and sensitive-line hei8834 at the third leaf stage.

To analyse the function of the CRPs, we determined the biological processes of these protein groups under chilling stress. We first examined the biological processes of shared responsive proteins (three upregulated and four downregulated) in KR701 and hei8834 at the first leaf and third leaf stage, suggesting that those proteins are related to the liquid transport and localization and ion transport pathways (Figure S6C,D). Except for those shared proteins (Figure 2D and Figure S6B), 618 proteins (180 shared, 214 KR701 specific and 224 hei8834 specific) specifically responded to chilling stress at the first leaf stage. By contrast, the number of CRPs was significantly decreased at the third leaf stage (only 27% of the first leaf stage, 169 CRPs, 9 shared, 123 KR701 specific and 37 hei8834 specific) (Figure 2C,D). Thus, the seedling stage, particularly the first leaf stage, is important for maize resistance to chilling stress. We next assessed the protein group functions of the CRPs at the first leaf and third leaf stage (Table S7). At the first leaf stage, 214 CRPs (164 upregulated and 50 downregulated) were specifically linked to the chilling response in KR701 lines, mainly functioning in photosynthesis and various hormone-mediated signalling pathways (particularly the ABA signalling pathway), and a small number of them were related to cell wall organization and catabolic pathways (Figure 3A,D). However, 225 CRPs (168 upregulated and 56 downregulated) specifically responding in hei8834 were mainly focused on cell wall organization, biogenesis and catabolic processes, ATP biosynthesis-related processes and hormone (only auxin) transport pathways (Figure 3B,E). Interestingly, at the third leaf stage, the biological processes of most CRPs (71 upregulated and 50 downregulated) in KR701 were the defence response and cell wall organization and metabolic pathways, a scenario similar to that of hei8834-specific CRPs at the first leaf stage (Figure 3G,J). By contrast, the CRPs were reduced significantly, and only 35 CRPs (6 upregulated and 29 downregulated) were specific to hei8834 at the third leaf stage compared with those at the first leaf stage, and these CRPs were involved in the photosynthesis pathway (Figure 3H,K). These proteomics results revealed entirely different situations between the chilling-tolerant-line KR701 and chilling-sensitive-line hei8834 under chilling stress. From the first leaf stage to the third leaf stage, the chilling-sensitive-line hei8834 skipped an essential biological process that induced ABA biosynthesis and signal transduction and proceeded directly to cell wall biogenesis and catabolic processes at the first leaf stage to confront chilling stress. These results further suggested that the proteomic patterns of ABA biosynthesis and signal transduction at the first leaf stage are important for maize resistance to chilling stress. We also examined the biological processes in which these chilling-responsive proteins are involved in both KR701 and hei8834 at the first leaf and third leaf stage, respectively. Proteome data suggested that these CRPs belong to fatty acid biosynthesis and metabolism, cinnamic acid biosynthesis and metabolism, lipid biosynthesis and localization pathways at the first leaf stage (151 overlapping upregulated and 29 overlapping downregulated proteins) and lipid localization and transport processes and peptidase activity at the third leaf stage (five overlapping upregulated, four overlapping downregulated and two KR701-specific upregulated and hei8834-specific downregulated proteins), suggesting that the biological processes related to organic acid- and lipid-related pathways also participate in the maize response to chilling stress (Figure 3C,F,I,L). However, these biological processes may not have been specifically responsible for maize resistance to chilling stress in our study.

### 3.4. Weighted Gene Coexpression Network Analysis (WGCNA)

**Figure 4:**
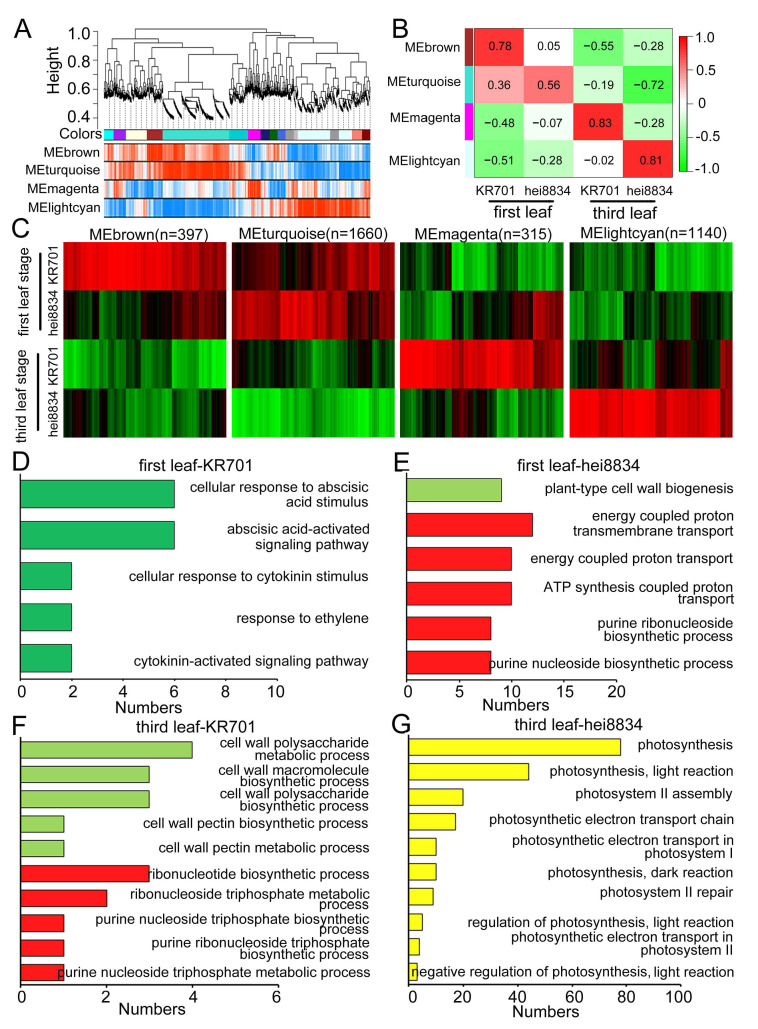
Network analysis of chilling stress in maize seedlings. (**A**) Coexpression modules identified using WGCNA. (**B**) Division of representative modules. At the first leaf stage, the change patterns of the tolerant-line KR701 were represented by the “MEbrown” module, and those of the sensitive-line hei8834 were represented by the “MEturquoise” module. At the third leaf stage, the change patterns of the tolerant-line KR701 were represented by the “MEmagenta” module, and those of the sensitive-line hei8834 were represented by the “MElightcyan” module. (**C**) Heatmap of the relative expression of 6631 genes in four representative stage-specific modules across all samples. (**D**–**G**) GO functional enrichment analysis of representative modules.

To further ensure that the ABA-related signalling pathway is critical for maize to resist chilling stress exclusively at the first leaf stage, we further evaluated our proteomics data using WGCNA (Table S8). We calculated the expression relationship among proteins to identify the modules of proteins with similar expression patterns and constructed the regulatory network among protein modules to determine the key regulatory proteins in the chilling stress response of maize. For the network conforming to a scale-free distribution, the power of the soft threshold was selected to make R^2^ of the scale-free network map reach 0.8 with the mean connectivity below 100 (Figure S7A,B). The R-square value acquired by the processing of the power value was approximately 0.8 (Figure S7C). Hierarchical clustering was conducted according to the similarity in gene expression, yielding the gene cluster tree, whose branches represent different gene modules labelled with different colours (Figure 4A). Simultaneously, the weighted correlation coefficient of genes was used to draw a topological overlap matrix (TOM) plot (Figure S7D) to classify genes according to their expression patterns: genes with similar patterns were categorized into the same module, and the representative modules were presented according to the *Pearson* correlation coefficient of modules and different treatments.

Four protein coexpression modules were modelled and related to KR701 and hei8834 at the first leaf and third leaf stages, respectively (Figure 4B, Table S8). We further clustered the proteins for each module, which exhibited distinct discrepancies among the protein groups (Figure 4C). GO enrichment analysis showed that at the first leaf stage of chilling stress, the representative module of KR701 was mainly related to hormone signalling responses, including ABA-, cytokinin- and ethylene-related pathways (Figure 4D), and that of hei8834 was mainly involved in cell wall biogenesis processes and ATP biosynthesis-related processes (Figure 4E). At the third leaf stage, the protein module of KR701 mainly participated in several cell wall anabolism and ATP synthesis and metabolic pathways (Figure 4F), and that of hei8834 was fully functional in the photosynthesis pathway (Figure 4G). Surprisingly, the two results obtained from two distinct algorithms (WGCNA for modules and CRPs for individual proteins) exhibited the same observation that the chilling-sensitive-line hei8834 skipped the ABA-related hormone signalling process at the first leaf stage compared with the chilling-tolerant-line KR701. These results further suggested that cell wall-related biological and metabolic processes, which would occur at the third leaf stage instead of the first leaf stage, are also crucial for maize to respond to chilling stress. As expected, the WGCNA results further confirmed that the ABA-related signalling pathway is important in maize resistance to chilling stress at the first leaf stage.

### 3.5. The Important Role of ABAin KR701 Subjected to Chilling Stress at the First Leaf Stage

**Figure 5:**
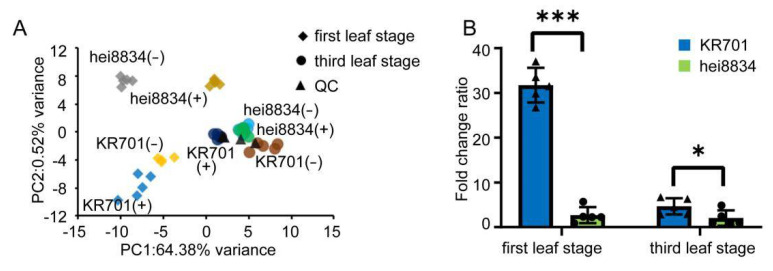
Metabolic profiling of maize plants subjected to chilling stress. (**A**) PLS analysis shows the divergence of the respective metabolomes in response to chilling stress. Different colors represent different samples. QC: quality control samples. (**B**) Changes in the endogenous ABA content. The y-axis is the fold change relative to the corresponding control: +chilling/−chilling. The data are expressed as the means of five replicates ±SD. * and *** denote levels of significance at *p* < 0.05 and *p* < 0.001, respectively.

To confirm the molecular patterns indicated by the CRP and WGCNA results showing that the ABA response process enhances the chilling resistance of maize seedlings at the first leaf stage, metabolomic analysis was performed. Partial least squares analysis (PLS) was performed to gain an overview of the different sample distributions for metabolomic responses (Figure 5A). Compared to control, the metabolite levels of KR701 and hei8834 changed markedly at the first leaf stage, while the changes were small at the third leaf stage under chilling stress The above results again show that the first leaf stage may be more critical for the mechanistic study of chilling stress than the third leaf stage. To further analyse the abundance profiles of ABA in the different samples, we analysed the fold change ratio of the endogenous ABA content after chilling stress (Figure 5B). After chilling stress, the endogenous ABA content of each sample increased. Interestingly, the endogenous ABA content increased 32-fold in the chilling-tolerant inbred line KR701 after chilling stress at the first leaf stage. These results are consistent with the observed increase in the expression of ABA biosynthetic proteins, suggesting that ABA synthesis may play a key role in the response to chilling stress for the tolerant-line KR701 at the first leaf stage.

## 4. Discussion

Chilling stress severely affects the growth and development of maize, and is the key factor for maize yield, particularly in high latitude areas [72,73,74]. Because the mechanism by which maize resists chilling stress is largely unknown, understanding the related proteome response patterns and protein functions is critical for breeding maize cultivars. In previous studies, the seedling stage was the main stage for chilling injury of maize [75,76,77]. Therefore, in this study, we selected two maize inbred lines (chilling-tolerant-line KR701 and chilling-sensitive-line hei8834) and investigated their proteome changes in different seedling stages (first leaf stage and third leaf stage).

**Figure 6:**
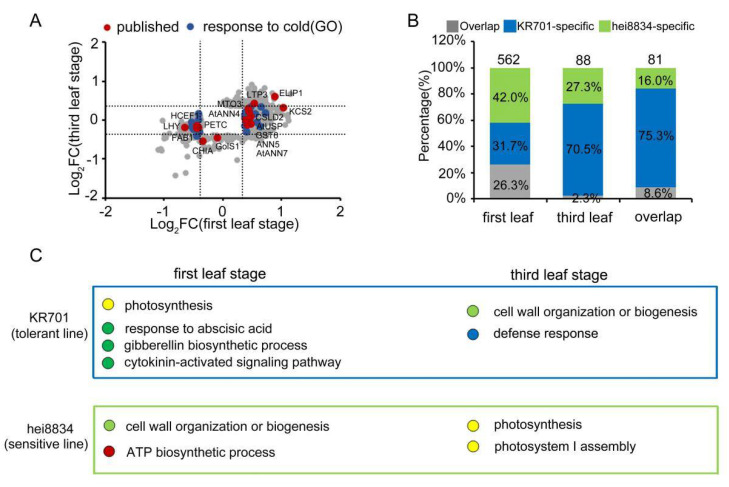
Regulatory module of maize seedlings under chilling stress. (**A**) Analysis of the distribution and related functions of CRPs at the first leaf and third leaf stages. Red spots: published chilling-related proteins. Blue spots: GO predicted chilling-related proteins. Grey spots: other CRPs. (**B**) Ratio of stage-specific CRPs at the first leaf stage and third leaf stage. Grey square: KR701- and hei8834-overlapping CRPs. Blue square: KR701-specific CRPs. Green square: hei8834-specific CRPs. (**C**) Diagram showing the key signalling events during the first leaf stage to the third leaf stage. Blue box: KR701-specific response. Green box: hei8834-specific response.

In this study, 19,717 maize proteins were identified, and the expression of 37% (7290/19717) of them was quantified under chilling stress, which significantly higher than previously quantified proteins [44,46]. According to previous reports and predictive GO analyses (Figure 6A), 16 published chilling-responsive proteins (red spots) [78,79,80,81,82,83,84,85,86,87,88,89,90,91,92] and 22 predicted chilling-responsive proteins (blue spots) were quantified in our proteome analyses. Interestingly, most of the published chilling-related proteins (75%) or predicted chilling-related proteins (96%) are only responsive at the first leaf stage but not at the third leaf stage, further supporting our suggestion that the first leaf stage is the key time point for maize resistance to chilling stress. More importantly, the published proteins and predicted proteins accounted for only a few portions (5%) in our chilling-responsive proteome. In our proteomic analyses, 562 proteins exhibited similar expression patterns under chilling and responded only at the first leaf stages as the published proteins, indicating they may also have important functions in the maize response to chilling stress (Figure 6B). Additionally, many proteins were responsive at both the first leaf and third leaf stages. The functions of these proteins warrant further study to identify novel mechanisms of maize resistance to chilling stress.

As expected (Figure 6C), the proteomics analyses exhibited distinct patterns between the chilling-tolerant-line KR701 and chilling-sensitive-line hei8834. This result suggested that after chilling stress, KR701 undergoes hormone-mediated signalling pathways, particularly the ABA signalling response process at the first leaf stage and the cell wall organization, biogenesis and catabolic response process at the third leaf stage. By contrast, chilling-sensitive hei8834 skips the ABA response process and proceeds directly to cell wall organization, biogenesis and catabolic-related processes at the first leaf stage and photosynthetic processes at the third leaf stage, causing the chilling-responsive proteins to be markedly reduced in hei8834. These results were further confirmed by WGCNA, suggesting that the proteome patterns of the ABA response process at the first leaf stage are crucial for maize resistance to chilling stress.

We further analysed chilling-responsive proteins at the first leaf stage and found that a few ABA synthesis and signal transduction proteins were upregulated only in chilling-tolerant KR701 but not in chilling-sensitive hei8834 at the first leaf stage. Because both KR701 and hei8834 had undergone a cell wall-related process but at different developmental stages, ABA synthesis and the related signal transduction might have been blocked in hei8834 during chilling stress. Thus, without a delayed effect or subsidiary of ABA signal transduction, hei8834 directly launched the cell wall organization process at the first leaf stage but not at the third leaf stage (as KR701). This result indicated that the chilling-resistance activities of hei8834 may be reduced by the deficiency of the subsidiary response or excessive energy consumption of cell wall synthesis at the premature stage (the first leaf stage). By contrast, the chilling-tolerant-line KR701 delayed the cell wall organization process at a later stage (the third leaf stage). 

Most previous physiological studies on chilling stress resistance have focused on the third leaf stage of maize, and only a few have mentioned the first leaf stage [8,39]. Based on the proteome patterns of KR701 and hei8834, we suggest that the first leaf stage is also critical for maize resistance to chilling stress. Our proteomics results further revealed that most published chilling-related proteins were primarily responsive to chilling stress at the first leaf stage but not at the third leaf stage. The physiological properties and protein functions of maize confronting chilling stress specifically at the first leaf stage warrant further investigation. 

## 5. Conclusions

Through comprehensive physiological, proteomics and hormonal analyses of different maize inbred lines (tolerant-line KR701 and sensitive-line hei8834) at different seedling stages (first leaf stage and third leaf stage) under chilling stress, we revealed several signalling proteins and pathways in response to chilling stress and that the phytohormone ABA response pathway may be critical for maize chilling resistance. Meanwhile, we found that the first leaf stage may be more suitable and important critical for the mechanistic study of chilling stress than the third leaf stage. In the future, different maize genetic materials are required to uncover more specific information. This study contributes to better understanding of the molecular mechanisms in the response of maize to chilling stress that may improve maize chilling tolerance and yield in the field.

## Data Availability

The data that support the findings of this study are available in the Supplementary Material section of this manuscript. The mass spectrometry proteomics data have been deposited to the ProteomeXchange Consortium via the PRIDE partner repository with the dataset identifier PXD023426.

## Supplementary Materials

The following supporting information can be downloaded at: https://www.mdpi.com/article/10.3390/cells11081321/s1, Figures S1–S7, Tables S1–S8. Figure S1. Changes in physiological traits of maize seedlings after chilling stress at the first leaf stage. Figure S2. Changes in physiological traits of maize seedlings after chilling stress at the third leaf stage. Figure S3. Display of the main data identified by proteomics. Figure S4. Sample correlation of proteomics data. Figure S5. Summary of the proteomics data. Figure S6. Co-expression analysis of CRPs. Figure S7. Assessment of soft thresholds used in WGCNA to generate coexpression networks. Table S1.Physiological responses induced by chilling stress in maize inbred lines KR701 (chilling tolerant) and hei8834 (chilling sensitive) at the first leaf and third leaf stage. Table S2. Basic profiles of proteomics data. Table S3. Protein abundance of the tolerant line KR701 and sensitive line hei8834 at the first leaf and third leaf stage. Table S4. The average abundance of proteins in different materials from the tolerant line KR701 and sensitive line hei8834 at the first leaf and third leaf stage. Table S5. The fold change ratio (abundance) of proteins in the tolerant line KR701 and sensitive line hei8834 at the first leaf and third leaf stage. Table S6. The log2 (fold change ratio) of responsive proteins at the first leaf and third leaf stage. Table S7. Distribution and GO enrichment analysis of CRPs. Table S8. Division of representative modules.
