# Supplementary material for: Comparative Proteomics Combined with Morphophysiological Analysis Revealed Chilling Response Patterns in Two Contrasting Maize Genotypes
Source: Cells. 2022 Apr 13;11(8):1321. doi: 10.3390/cells11081321 (PMC9024610; doi:10.3390/cells11081321)
Supplement: Supplementary file 1 [file cells-11-01321-s001.zip › cells-1669641 supplementary conversion.pdf]

**Supplemental Data**

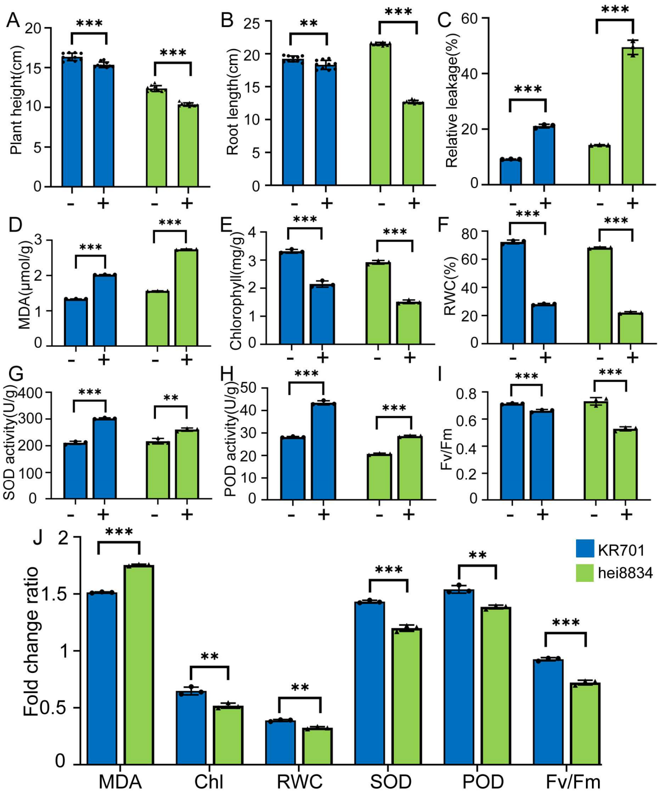


**Figure S1. Changes in physiological traits of maize seedlings after chilling stress at the first leaf stage. (A-I)** Changes in the measured values of ten physiological traits after chilling stress. Different stress treatments: -: uniformly growing seedlings were grown at normal condition for 24 h. +: uniformly growing seedlings were grown at 4°C for 24 h. **(J)** The fold change ratio of six representative traits. Fold change ratio: +chilling/-chilling. Blue square: KR701. Green square: hei8834. The plant height and root length data are the means ± SD of ten replicates. Other trait data are means ± SD of three replicates. ** and *** denote levels of significance at $p < 0.01$ and $p < 0.001$, respectively.

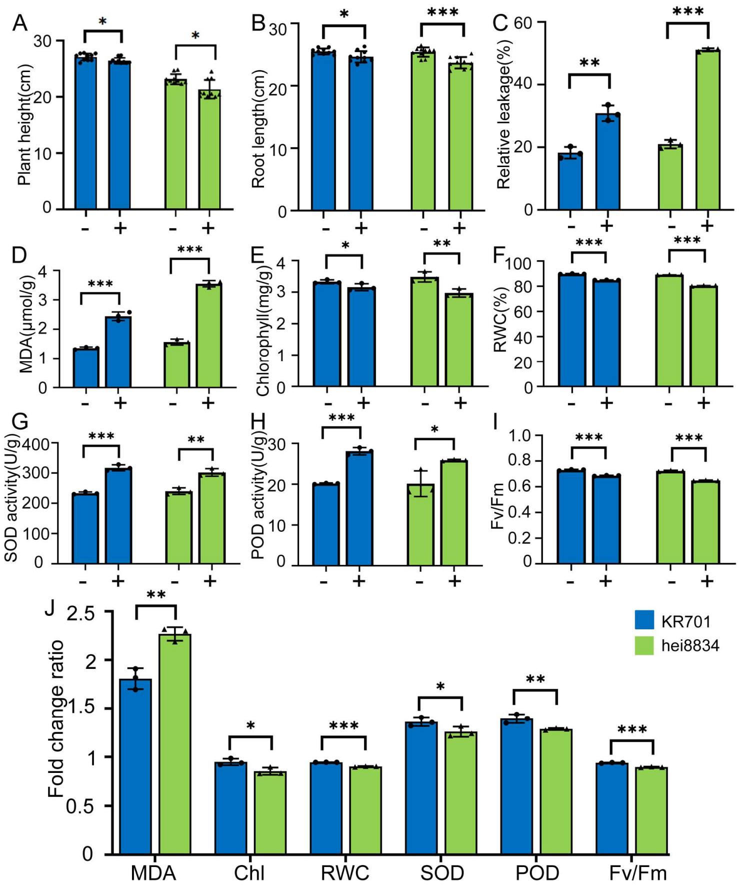


**Figure S2. Changes in physiological traits of maize seedlings after chilling stress at the third leaf stage. (A-I)** Changes in the measured values of ten physiological traits after chilling stress. Different stress treatments: -: uniformly growing seedlings were grown at normal condition for 24 h. +: uniformly growing seedlings were grown at 4°C for 24 h. **(J)** The fold change ratio of six representative traits. Fold change ratio: +chilling/-chilling. Blue square: KR701. Green square: hei8834. The plant height and root length data are the means ± SD of ten replicates. Other trait data are means ± SD of three replicates. *, ** and *** denote levels of significance at $p < 0.05$, $p < 0.01$ and $p < 0.001$, respectively.

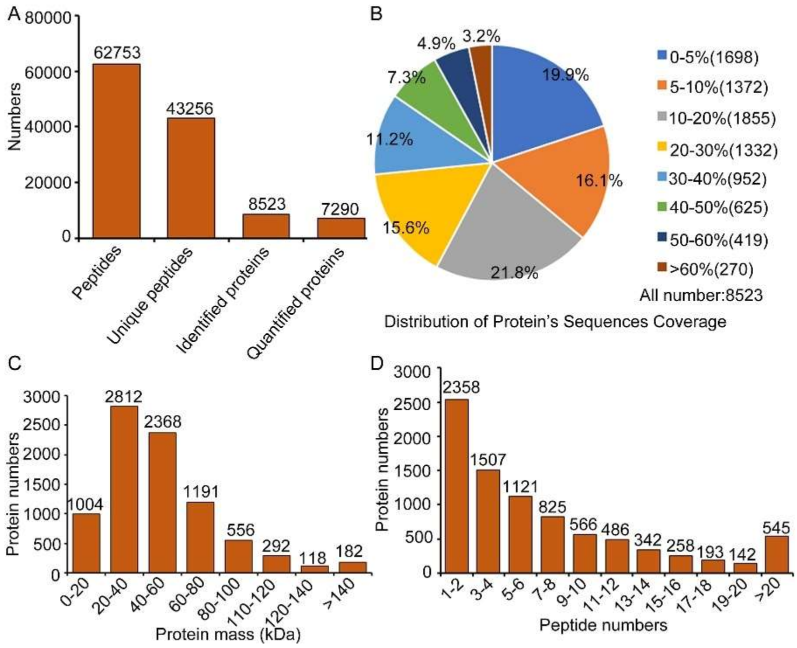


**Figure S3. Display of the main data identified by proteomics. (A)** The proportional distribution of the number of peptides and proteins identified in proteomics by searching the maize database. **(B)** The distribution of protein sequence coverage. **(C)** The distribution of the identified protein size. **(D)** Proportional distribution of the number of identified peptides.

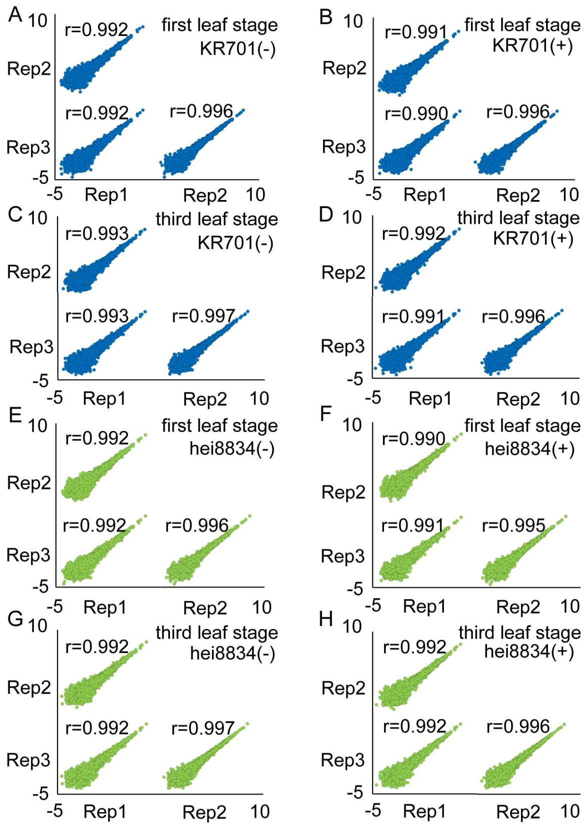


**Figure S4. Sample correlation of proteomics data.** The repeatability of the experiment was verified by data correlation analysis among three replicates. *Pearson* correlation coefficient analysis was used. **(A, B, C, D)** Tolerant line KR701. **(E, F, G, H)** Sensitive line hei8834. (-): uniformly growing seedlings were grown at normal condition for 24 h. (+): uniformly growing seedlings were grown at 4°C for 24 h.

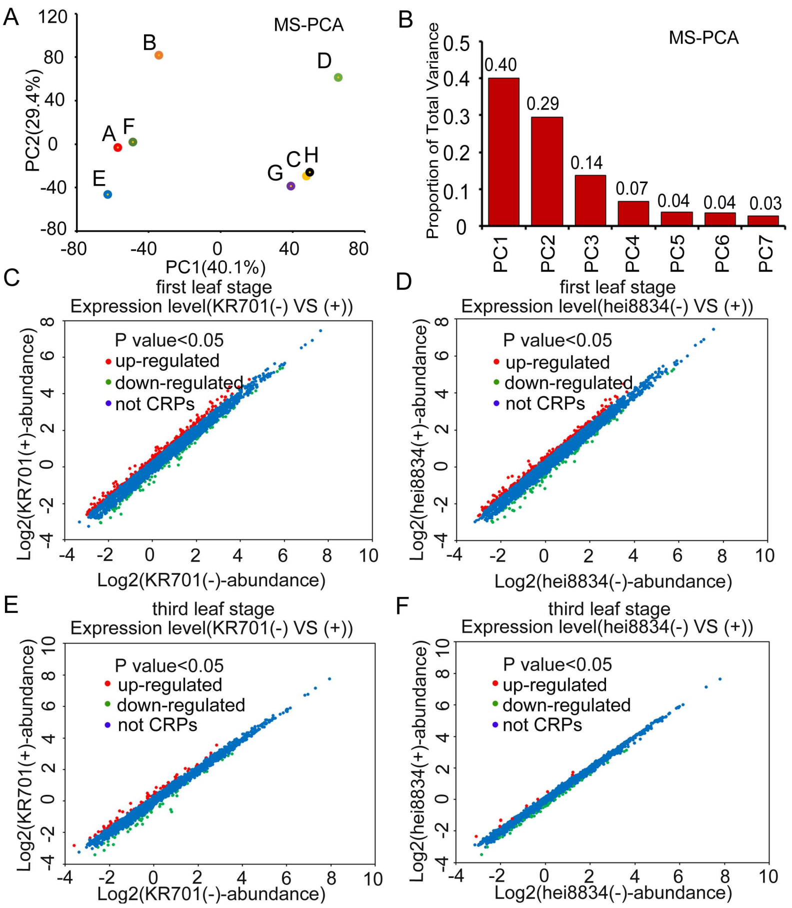


**Figure S5. Summary of the proteomics data. (A)** Principal component (PCA) analysis. Letters A-D: KR701(-), KR701(+), hei8834(-) and hei8834(+) at the first leaf stage. Letters E-H: KR701(-), KR701(+), hei8834(-) and hei8834(+) at the third leaf stage. **(B)** The proportion of the total variance of each principal component. **(C, D, E, F)** The overall distribution of protein abundance. The red, green and blue dots represent the upregulated CRPs, downregulated CRPs and non-CRPs, respectively. (-): uniformly growing seedlings were grown at normal condition for 24 h. (+): uniformly growing seedlings were grown at 4°C for 24 h.

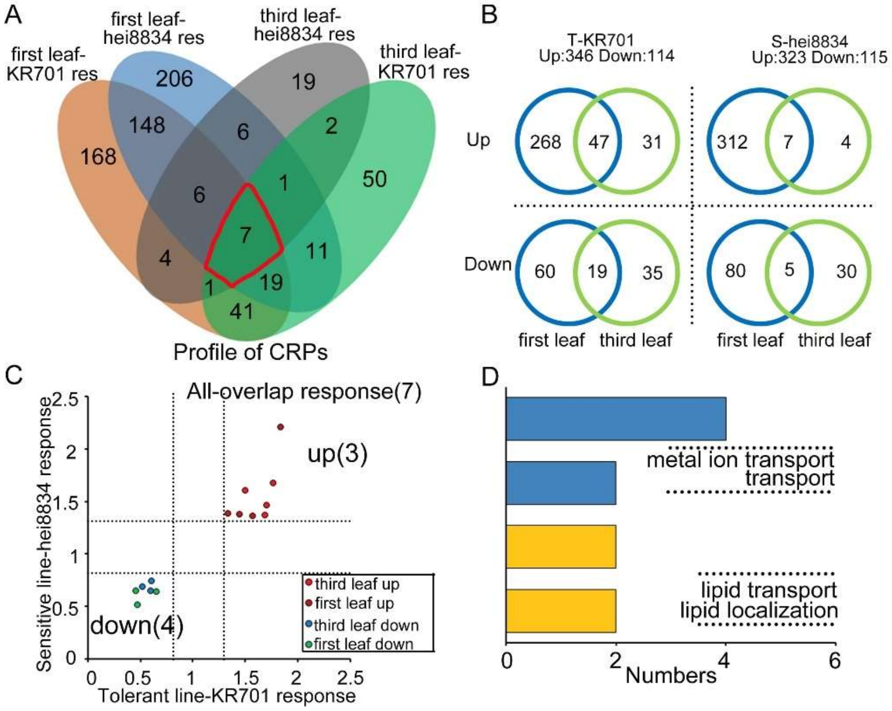


**Figure S6. Co-expression analysis of CRPs. (A)** Profile of CRPs. **(B)** Classification of CRPs. **(C)** The specific distribution of all overlapping CRPs. **(D)** GO enrichment analysis of all overlapping CRPs.

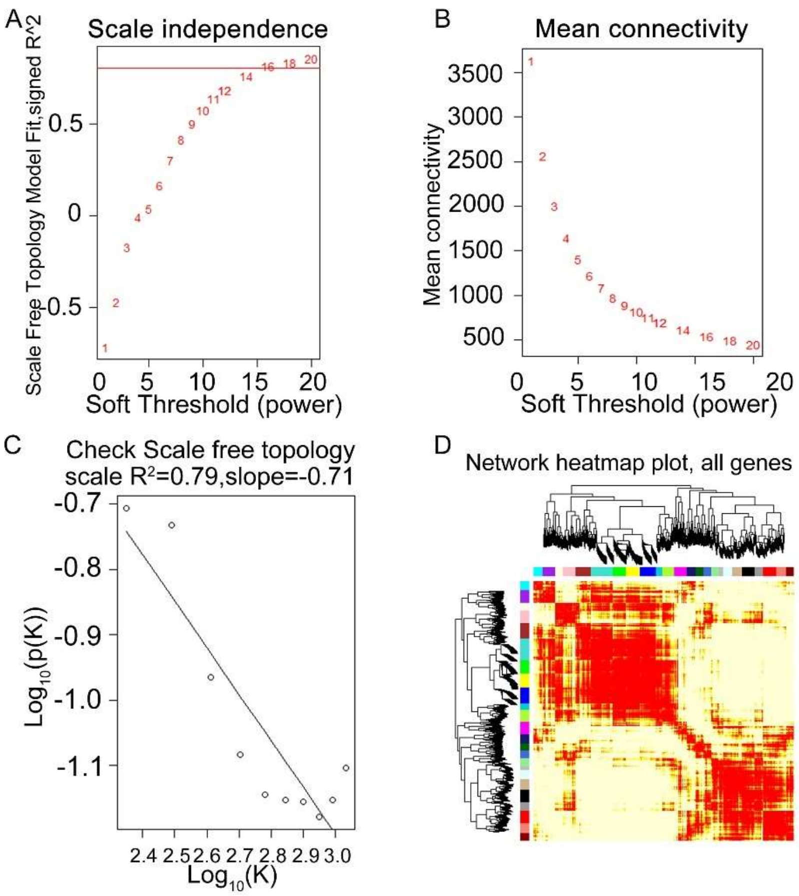


**Figure S7. Assessment of soft thresholds used in WGCNA to generate coexpression networks. (A)** Scale independence for the coexpression network. The red line shows the position where R^2 reaches 0.8. **(B)** Mean connectivity in the coexpression network. **(C)** Reliability verification of power selection. **(D)** Visualized gene network (TOM plot).

**Supplemental Table S1**

Physiological responses induced by chilling stress in maize inbred lines KR701 (chilling tolerant) and hei8834 (chilling sensitive) at the first leaf and third leaf stage.

**Supplemental Table S2**

Basic profiles of proteomics data.

**Supplemental Table S3**

Protein abundance of the tolerant line KR701 and sensitive line hei8834 at the first leaf and third leaf stage.

**Supplemental Table S4**

The average abundance of proteins in different materials from the tolerant line KR701 and sensitive line hei8834 at the first leaf and third leaf stage.

**Supplemental table S5**

The fold change ratio (abundance) of proteins in the tolerant line KR701 and sensitive line hei8834 at the first leaf and third leaf stage.

**Supplemental Table S6**

The $\log_2$ (fold change ratio) of responsive proteins at the first leaf and third leaf stage.

**Supplemental Table S7**

Distribution and GO enrichment analysis of CRPs.

**Supplemental Table S8**

Division of representative modules.
